# Parkinson’s Neuropathology Puzzle: A Systematic Review Uncovering the Pathological Culprits Behind the Neurological Disease

**DOI:** 10.7759/cureus.44353

**Published:** 2023-08-29

**Authors:** Abdelrahman Abaza, Aneeque Jamil, Sai Dheeraj Gutlapalli, Marya Ali, Mrinal J. P. Oble, Shamsun Nahar Sonia, Sherie George, Srushti R Shahi, Zahra Ali, Safeera Khan

**Affiliations:** 1 Pathology, California Institute of Behavioral Neurosciences and Psychology, Fairfield, USA; 2 Internal Medicine, California Institute of Behavioral Neurosciences and Psychology, Fairfield, USA; 3 Internal Medicine, Richmond University Medical Center Affiliated With Mount Sinai Health System and Icahn School of Medicine at Mount Sinai, Staten Island, USA; 4 Internal Medicine Clinical Research, California Institute of Behavioral Neurosciences and Psychology, Fairfield, USA; 5 Psychiatry, Nishtar Medical University, Multan, PAK; 6 Medicine, Kempegowda Institute of Medical Sciences and Research Centre, Bengaluru, IND; 7 General Medicine, Pinderfields Hospital, Leeds, GBR; 8 Medicine, St. Martinus University Faculty of Medicine (SMUFOM), Willemstad, CUW; 9 Medicine, Bolan Medical College, Quetta, PAK; 10 Medicine, California Institute of Behavioral Neurosciences and Psychology, Fairfield, USA

**Keywords:** parkinson's pathological stages, parkinson's neuropathology, parkinsonian disorders' subdivisions, parkinson's pathophysiology, parkinson's disease

## Abstract

Being one of the most prevalent progressive neurodegenerative disorders (falling second only to Alzheimer’s disease) with a clinical pattern affecting millions of lives all over the world, Parkinson’s disease (PD) has never failed to attract a formidable interest from the vast majority of neurologists and researchers worldwide. This review article will analyze the pathophysiology, etiology, genetics, and pathological stages of Parkinson’s disease with their corresponding clinical sequels. A review article was conducted using research databases including PubMed, PubMed Central, Springer, and Elsevier. The research articles reviewed using databases were written in English, German, Japanese, and Chinese and published within the preceding 50 years. Based on the article’s findings, we concluded that Parkinson’s disease is a progressive disorder with a variety of motor and non-motor symptoms that are influenced by a cascade of pathological neuronal abnormalities such as Lewy neurites and Lewy bodies that gradually build up with an eventual consequence of neurodegeneration of dopamine-secreting neurons. Multiple genetic mutations, pathophysiological events, and environmental factors act as the foundation to initiate that cascade.

## Introduction and background

Even though the first Parkinson’s disease (PD) pathological illustrations were as early as multiple centuries before Christ (BC), however, the best medical characterization of PD dates back to 1817, when James Parker wrote his paper "Essay on the Shaking Palsy," a detailed description of six cases of PD’s patients outlaying the unique clinical picture of this neurodegenerative disorder [1,2]. Further clinical delineation of the pathological progression of PD was performed by Richer and Meige in 1895 to demonstrate the morphology of the progression of PD [3]. In addition, Greenfield and Bosanquet provided the most comprehensive description of PD, illustrating the pathological brain stem lesions characterizing PD [4]. The breakthrough regarding medical therapy for PD was the introduction of Levo-Dopa to overcome the bothersome symptoms of PD [5].

Clinically, Parkinson’s disease is a progressive neurodegenerative disorder presenting with many symptoms, including resting tremors, bradykinesia, muscular rigidity, a shuffling gait, a masked face, and postural instability. In addition, as the disease progresses, patients suffer from non-motor symptoms such as depression, rapid eye movement (REM) sleep disorders, gradual loss of sense of smell, dementia, and autonomic dysfunction [6].

Pathologically, Parkinson’s disease is defined by the loss of dopamine-secreting neurons, especially the ventrolateral cells in the substantia nigra pars compacta, which project to the basal ganglia, causing dopamine denervation to the striatum, which controls the coordination of motor and action planning. In addition, α-synuclein aggregates in the neurons, producing the characteristic Lewy bodies, which can be found in other pathological conditions such as Lewy body dementia, multiple system atrophy, and progressive supranuclear palsy [7].

With these remarkable findings over the years, multiple research projects were conducted to provide reliable information regarding the etiology, pathogenesis, neuropathology, clinical picture, diagnostic measures, and therapeutic interventions that tremendously influenced our understanding of the whole picture of one of the most prevalent neurodegenerative disorders ever. This review article focuses on Parkinsonism’s subdivisions, genetics, pathophysiology, and pathological stages in correlation with the clinical syndrome.

## Review

Methods

Implementing the Preferred Reporting Items for Systematic Reviews and Meta-Analyses (PRISMA) design supported our vision to perform a transparent and in-depth systematic review.

Search Design

The research team used multiple databases to provide pertinent data regarding the research design, including PubMed, PubMed Central, Springer, and Elsevier. The keywords implemented during our research were "Parkinson’s disease history," "Parkinson’s neuropathology," "Parkinson’s pathophysiology," "Parkinson’s pathological stages," and "Parkinson’s clinical correlation."

Inclusion and Exclusion Criteria

A demonstration of the study design's inclusion and exclusion criteria is displayed in Table 1.

**Table 1:** Inclusion and exclusion criteria This table demonstrates the inclusion and exclusion criteria implemented during the selection process of review articles.

Inclusion criteria	Exclusion criteria
1. Papers released in the last 50 years	1. Papers released more than 50 years ago
2. Papers written in English, German, Japanese, and Chinese	2. Papers written in the other languages
3. Papers with a special focus on older adults and the geriatric population	3. Papers including children and young adult population
4. Papers with relevance to the topic discussed	4. Papers without any relevance to the topic
5. Published literature reviews and systematic reviews	5. Case studies, observational studies, randomized controlled trials, and grey literature

Analysis of Study Quality

Three authors conducted independent reviews of related papers to select relevant information for this review. Following the rapid scrutiny of the titles, abstracts that fit the inclusion criteria were further checked for the reliability of the ideas presented. Once reliability was assured, selected articles were entirely read to confirm the clarity and precision of the information discussed. Finally, 16 highly informative review articles were selected after meeting the criteria of the Scale for the Assessment of Narrative Review Articles (SANRA) checklist [8]. The SANRA scoring system ranges from zero to two for each standard of the checklist, with an overall score of ≥10 considered to be representative of a high-quality review article.

Table 2 shows the detailed quality of the review articles that met the criteria of the SANRA checklist.

**Table 2:** Scale for the Assessment of Narrative Review Articles (SANRA) checklist scores for the included review articles This table demonstrates the quality of the individual review articles based on the Scale for the Assessment of Narrative Review Articles (SANRA) checklist.

Publication	Elsworth [9]	Antony et al. [10]	Goetz [11]	Bekris et al. [12]	Koga et al. [13]	Beitz [14]	Puschmann [15]	Cacabelos [16]	Dauer and Przedborski [17]	Dorsey et al. [18]	Lotankar et al. [19]	Aryal et al. [20]	Titova et al. [21]	Stott et al. [22]	Irwin et al. [23]	Dickson [24]
Justification of the article's importance	2	2	2	2	2	1	2	1	2	2	2	1	2	2	2	2
Statement of concrete aims or formulation of questions	1	1	2	2	1	2	2	2	2	2	1	2	2	1	2	2
Description of the literature search	1	2	2	2	2	2	2	1	1	2	2	2	2	2	1	2
Referencing	2	2	2	1	2	2	2	2	2	2	1	2	2	1	2	2
Scientific reasoning	2	1	2	2	2	2	1	2	2	2	2	2	1	2	1	2
Appropriate presentation of data	2	2	2	2	2	1	2	2	2	2	2	1	2	2	2	2
Score	10	10	12	11	11	10	11	10	11	12	10	10	11	10	10	12

Data Extraction

Implementing Preferred Reporting Items for Systematic Reviews and Meta-Analyses (PRISMA) guidelines was crucial to data extraction. Review articles from different databases were picked through a highly selective process comprising multiple quality assessments to exclude irrelevant or deficient articles. Articles written in languages other than English were meticulously translated by two independent authors.

**Figure 1:**
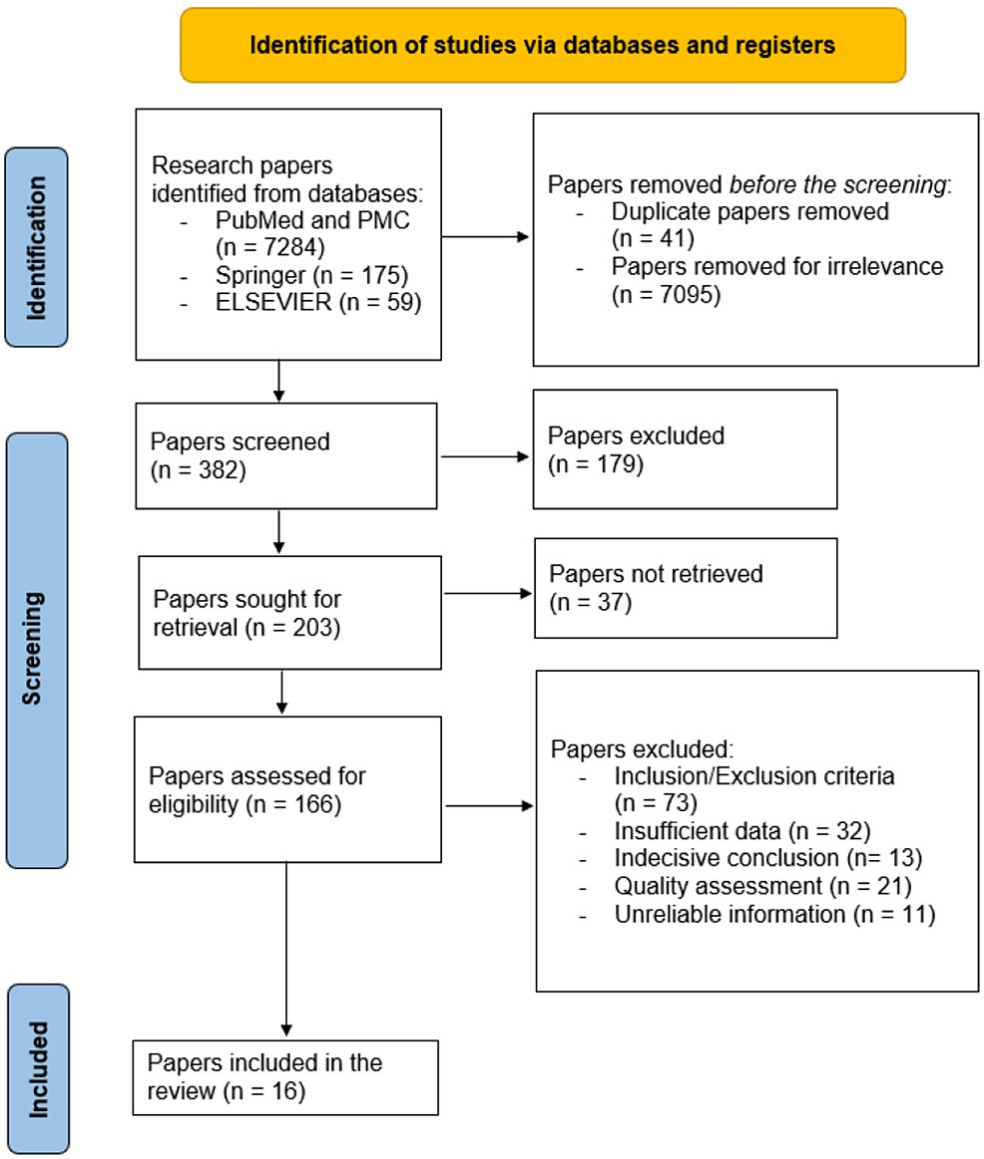
Preferred Reporting Items for Systematic Reviews and Meta-Analyses (PRISMA) flowchart data extraction process This image demonstrates the data extraction process based on Preferred Reporting Items for Systematic Reviews and Meta-Analyses (PRISMA) flowchart.

Figure 1 shows the data extraction process by implementing the PRISMA flowchart.

Results

During the first database approach, 7518 research papers were assessed. Of these papers, 41 were eliminated because of duplication, and 7095 were precluded due to irrelevance. Many research papers were screened based on the precision of the ideas presented, leading to the exclusion of 179 research papers and the retrieval of 203 research papers, which were subjected to further eligibility assessment based on multiple screening criteria that eventually ended with 16 research articles meeting all standards and being deservedly embraced in our review article.

Discussion

Being the second most common progressive neurodegenerative disorder only after Alzheimer's, PD is among the most prevalent neurological disorders, achieving a record of between 5 and 35 cases in every 100,000 individuals [25]. PD is characterized by variable clinical signs and symptoms, including the hallmark signs of generalized slowing of gross and fine motor function, cog-wheel muscular rigidity, asymmetric progressive resting tremors, shuffling short-stepped gait, and postural instability. It also presents with non-motor symptoms such as depression, constipation, REM sleep disorders, gradual hyposmia, dementia, and autonomic dysfunction [26]. Multiple factors predispose patients to develop PD, including smoking, toxic chemicals, heavy metals, repeated traumatic head injuries, and genetic factors. Other factors are considered protective, such as a vegetable- and fruit-rich diet, exercise, and caffeine [27].

**Figure 2:**
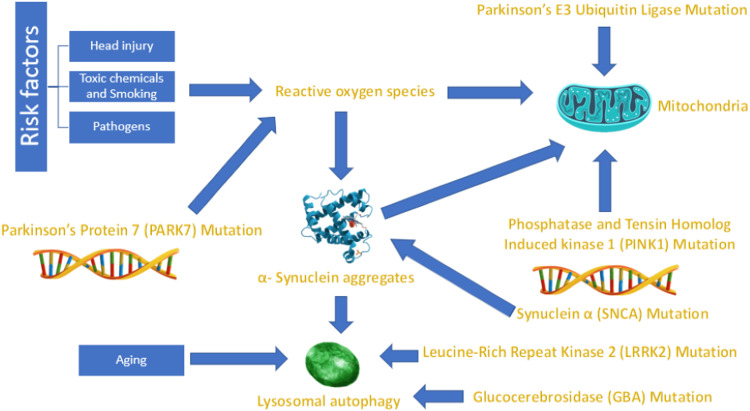
Pathophysiology of Parkinson's disease The image demonstrates the pathophysiological mechanisms that contribute to the development of Parkinson's disease. Source: Simon et al. [28].

The pathophysiology of PD can be explained in Figure 2 [28].

Parkinson's disease is closely associated with multiple genetic mutations occurring at the chromosomal level. These genetic mutations include synuclein α (SNCA), Parkinson's (PARKIN) E3 ubiquitin ligase, phosphatase, and tensin homolog-induced kinase one (PINK1), Parkinson’s protein seven (PARK7), glucocerebrosidase (GBA), and leucine-rich repeat kinase two (LRRK2). SNCA mutations are associated with the production of α-synuclein aggregates in neurons, which represent a significant component of Lewy bodies and are associated with a higher risk of developing Lewy body dementia, autosomal dominant (AD) PD, and sporadic PD [29]. PARKIN and PINK1 mutations predispose to failure of the breakdown of functionally impaired mitochondria by the lysosomal autophagy pathway, causing the piling up of functionally impaired mitochondria with loss of the turnover process of mitochondria that predisposes patients to early-onset autosomal recessive (AR) PD [30,31]. PARK7 mutations result in the failure of regulation of transcription factors that are responsible for the upregulation of different antioxidant functions, including glutathione production, resulting in the vulnerability of the cells against reactive oxygen species (ROS), which also predisposes to early-onset AR PD [32]. GBA mutations result in the failure of lysosomal glucocerebrosidase enzymes to break down glucocerebrosides, which accumulate inside cells, predisposing to both Gaucher disease and PD [33]. LRRK2 is a serine/threonine kinase consisting of two domains: guanosine triphosphatase (GTPase) in conjunction with a kinase domain. Mutations in the LRRK2 gene result in unregulated kinase activation, with resultant neurotoxicity associated with AD PD [34]. From the preliminary information, we can conclude that multiple genetic and chromosomal factors play a formidable role in the pathophysiology of PD.

Parkinsonian Disorders

Various pathological entities fall into the category of Parkinsonian disorders, and they are all characterized by the loss of dopamine-secreting neurons in the substantia nigra that project to the putamen in the striatum of the basal ganglia that represent the nigrostriatal pathway. What these neurons share in common is the deficiency in their ability to buffer intracellular calcium transients that can lead - up to their accumulation inside neurons - to the disturbance of the homeostasis of cells with the resultant loss of integrity of the membranes of the nucleus of these neurons and the liberation of histones that act as a stimulus to release aggregates of α-synuclein proteins, which represent the hallmark of Parkinsonian disorders [35-37].

**Figure 3:**
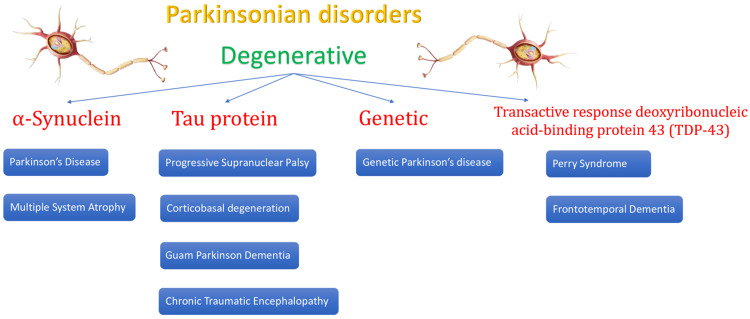
Degenerative causes of Parkinsonism This image reveals the impact of various degenerative processes on the development of Parkinson's disease. Source: Dickson [38].

Parkinsonian disorders can be subclassified as degenerative or non-degenerative. The degenerative causes of Parkinsonism are summarized in Figure 3.

**Figure 4:**
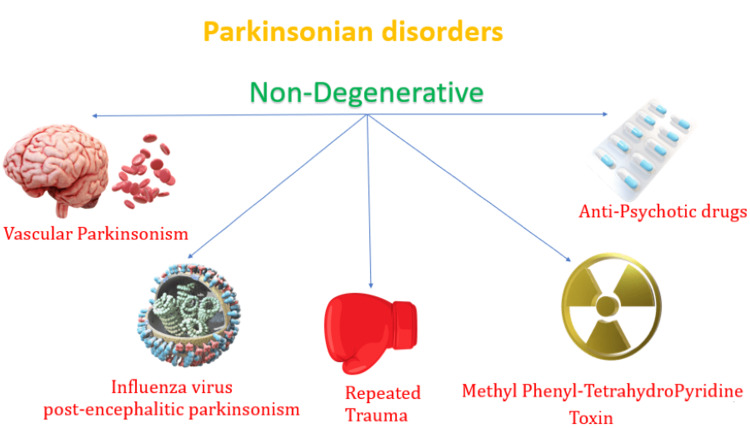
Non-degenerative causes of Parkinsonism This image demonstrates the impact of various non-degenerative processes on the development of Parkinson's disease. Source: Dickson [38].

Non-degenerative causes of Parkinsonism are summarized in Figure 4.

Pathological Stages of Parkinsonism

Pathological stages of Parkinson’s disease require extended duration to attain the full clinical scope of PD. As parkinsonism progresses, the neuronal lesions accumulate constantly, and remarkable changes happen in their respective allocations. These variations even form in asymptomatic patients before any actual physical or clinical findings appear on the surface and can be subclassified into six pathological stages based on the continuous emergence of specific inclusion bodies within the involved neurons, such as Lewy bodies and Lewy neurites, which are made of aggregates of α-synuclein proteins [39]. Some neurons exhibit more vulnerability than others against the aggregation of such proteins, which renders them particularly susceptible to progressive neurodegeneration. This selective vulnerability is displayed by highly pronounced neuronal damage to the somatomotor and visceromotor centers and other brain areas related to motor functions, such as the limbic system, with the relative sparing of somatosensory centers except for olfactory neurons. What these susceptible neurons share in common is that they are mostly projection neurons with relatively lengthy axons compared to their cell body size and absent or inadequate protection with myelin sheath, which further contributes to their lack of resistance against the development of Lewy bodies or Lewy neurites as PD progresses [40].

Stage 1: The first PD pathological findings almost always appear with the formation of isolated scattered Lewy neurites at the dorsal motor nucleus of the vagus nerve, adjoining intermediate reticular zone, olfactory bulb, anterior olfactory nucleus, and vasoactive intestinal peptide (VIP) secreting neurons within the enteric nervous system, with complete sparing of solitary and ambiguous nuclei of the vagus nerve [41].

**Figure 5:**
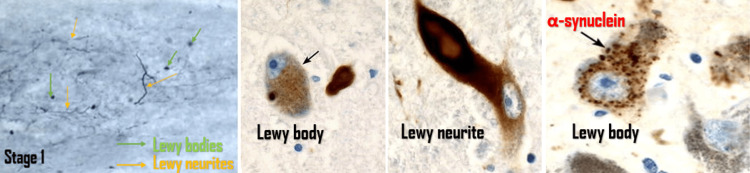
Lewy bodies and Lewy neurites in stage one of Parkinson's disease This image demonstrates the accumulation of Lewy bodies and Lewy neurites in stage one of Parkinson's disease (using α-synuclein immunoreactions in paraffin sections). The images were reproduced with permission from Professor Heiko Braak. Source: Braak et al. [42].

Figure 5 shows scattered Lewy bodies and Lewy neurites in pathological stage one of PD [42].

Stage 2: The pathological neuronal findings involve the medullary raphe nuclei, the gigantocellular reticular nucleus of the medullary reticular formation, and the pontine locus coeruleus nucleus. These nuclei together represent a pain regulation multisystem that inhibits sensory input relay nuclei. Furthermore, they regulate the excitability of premotor and motor neurons in the medulla oblongata and spinal cord. Stages 1 and 2 represent the presymptomatic stages of PD that are limited to the medullary and pontine tegmentum before the involvement of the substantia nigra, thus leading to the development of characteristic clinical signs and symptoms distinctive of PD [43].

Stage 3: The pathological neuronal findings extend further to involve the substantia nigra pars compacta of the midbrain, initially in the posterolateral nucleus, followed by the posterosuperior and posteromedial nuclei, followed by the involvement of the pedunculopontine nucleus of the tegmentum of the pons, the central nucleus of the amygdala, the hippocampus, the magnocellular nuclei of the basal forebrain, and finally the tuberomammillary nucleus of the hypothalamus. In stage 3, Lewy neurites and Lewy bodies shift from scattered inclusions to the appearance of highly crowded granular aggregates within the neurons, which marks the beginning of the transition from the presymptomatic to the clinically evident phase of symptomatic PD.

**Figure 6:**
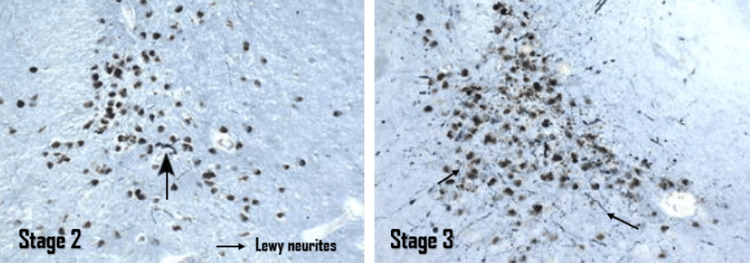
Lewy bodies and Lewy neurites in stages two and three of Parkinson's disease This image demonstrates the progression of accumulation of Lewy bodies and Lewy neurites in stages 2 and 3 of Parkinson's disease (using polyethylene glycol sections stained for Nissl material and immunoreactions for α-synuclein). The images were reproduced with permission from Professor Heiko Braak. Source: Braak et al. [42].

Figure 6 shows Lewy bodies and neurites in stages two and three of PD [42].

Stage 4: The pathological neuronal findings extend to the cerebral cortex to involve mainly the temporal mesocortex and entorhinal region of the allocortex, with further progression of the neurodegenerative process. At a certain point between stages 3 and 4, the classical characteristic signs of PD, such as resting tremors, bradykinesia, muscular rigidity, and shuffling gait, start to emerge [44].

Stage 5: The pathological neuronal findings extend further to involve the prefrontal cortex and the high-order sensory association zones.

Stage 6: The pathological neuronal findings extend further to involve the premotor and first-order sensory association zones and can even reach the primary sensory fields.

**Figure 7:**
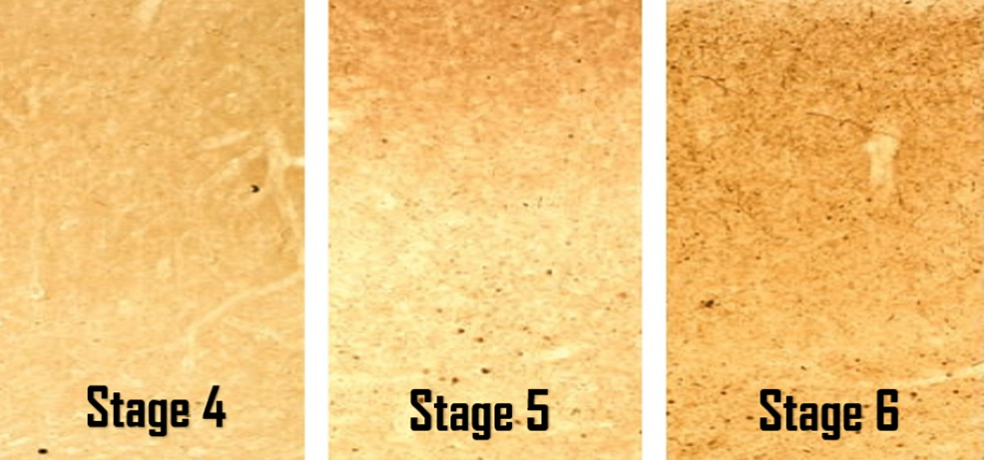
Lewy bodies and Lewy neurites in stages 4, 5, and 6 in Parkinson's disease This image demonstrates the progression of accumulation of Lewy bodies and Lewy neurites in stages 4, 5, and 6 of Parkinson's disease (using polyethylene glycol sections stained for immunoreactions for α-synuclein). The images were reproduced with permission from Professor Heiko Braak. Source: Braak et al. [42].

Figure 7 shows the progression of the pathological Lewy bodies and neurites in the entorhinal region of the allocortex in stages 4, 5, and 6 of PD [42].

During these late stages, the full clinical scope of PD is finally reached with the worsening of the motor symptoms, including severe resting tremors, gait disorders, frequent falling, postural instability, and restless leg syndrome, and the emergence of non-motor symptoms such as progressive dementia, depression, REM sleep disorders, autonomic dysfunction, delusions, hallucinations, and personality changes.

## Conclusions

Parkinson’s disease is a multisystem progressive α-synuclein protein-dependent neurodegenerative disorder. The pathological hallmark of PD is the α-synuclein protein aggregates that build up in the form of Lewy bodies and Lewy neurites in the neurons’ cell bodies and then march upwards, starting from medullary nuclei and involving the selectively vulnerable neurons of the pons, midbrain, hypothalamus, and eventually the cerebral cortex. Specific genetic mutations, pathophysiological events, and environmental factors, either individually or collectively, predispose to the initiation of the cascade of such pathological findings and eventually lead to cumulative neuronal degeneration that can be manifested by the characteristic clinical findings we can all observe in PD patients.
